# Supplementary figures and images for: Immunisation of chickens with inactivated and/or infectious H9N2 avian influenza virus leads to differential immune B-cell repertoire development
Source: Front Immunol. 2024 Oct 28;15:1461678. doi: 10.3389/fimmu.2024.1461678 (PMC11555566; doi:10.3389/fimmu.2024.1461678)

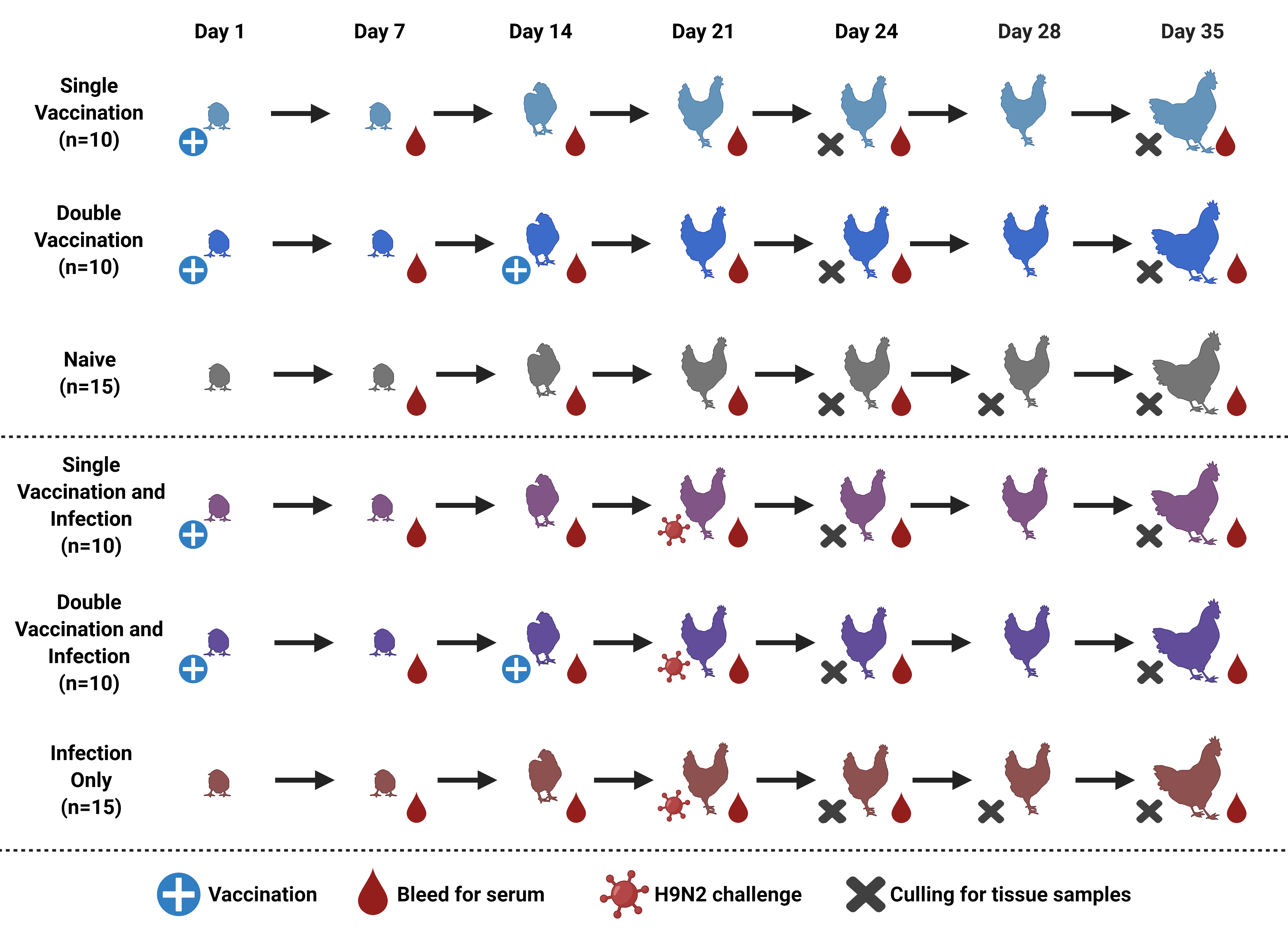

Supplement: Supplementary Figure 1 — Design of the H9N2 vaccination and infection experiment. Birds were split into 6 groups which received either an inactivated H9N2 vaccine at day 1, or both at days 1 and 14, or no vaccination. At day 21, half of the birds (belonging to all vaccination regime treatments) were infected with H9N2 avian influenza virus. Birds were then culled at days 24 (n=5 from all groups), day 28 (n=5 from the unvaccinated treatments), and at day 35 (n=5 from all groups). Blood and tissue samples were harvested and processed for subsequent analyses. Buccal and cloacal swab samples were collected from the infected birds with one pre-infection sampling and 10 other daily swabs after infection. [file Image1.jpeg]

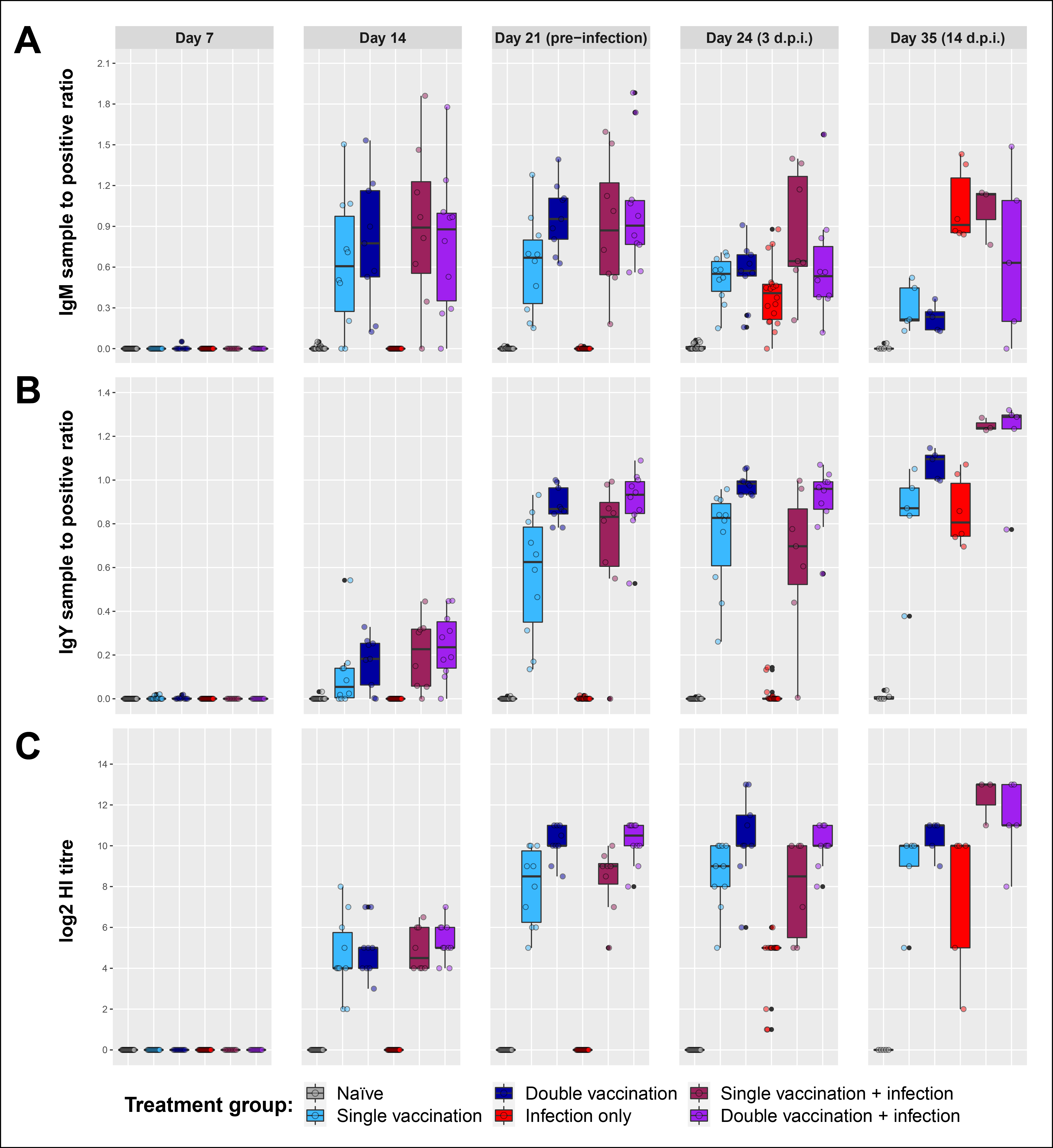

Supplement: Supplementary Figure 2 — H9N2-specific antibody levels and haemagglutination inhibition (HI) potential of sera in chickens following vaccination and infectious challenge. (A) IgM ELISA sample-to-positive ratios. (B) IgY ELISA sample-to-positive ratios. (C) HI titres of serum samples. [file Image2.jpeg]

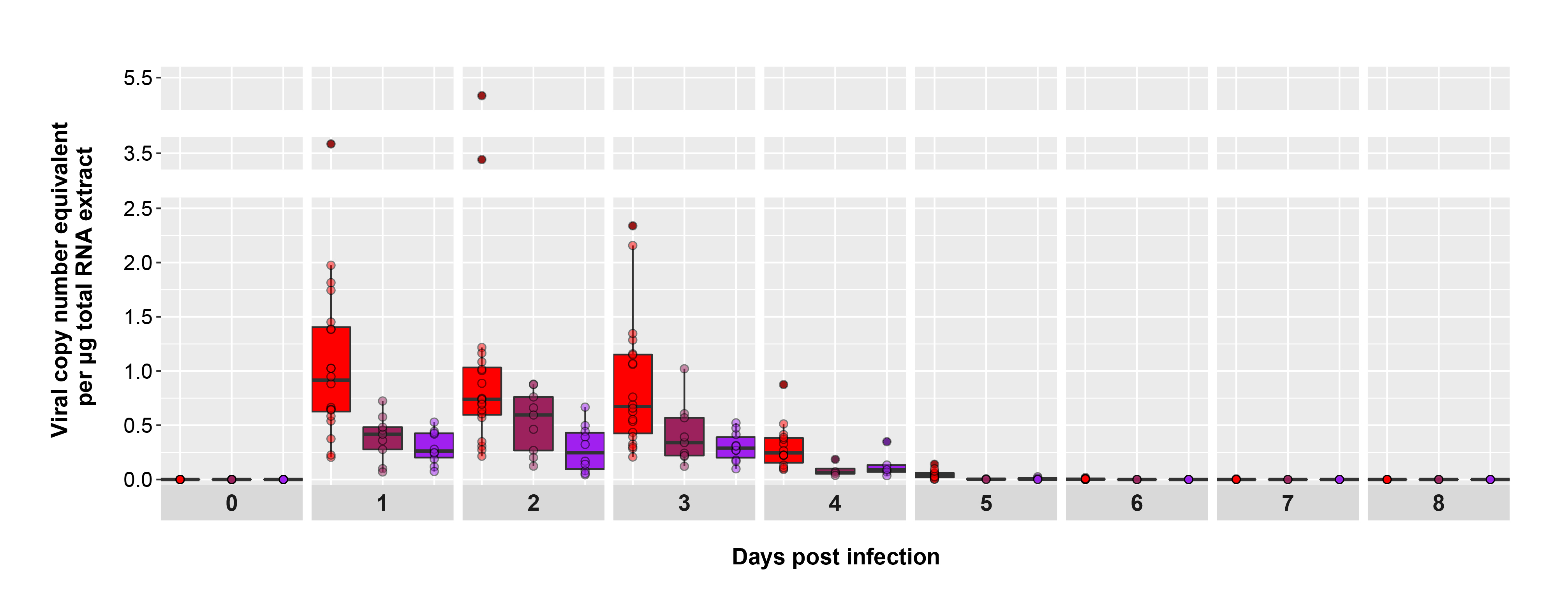

Supplement: Supplementary Figure 3 — H9N2 matrix gene qRT-PCR results of buccal swab samples. Viral copy number equivalent per μg total RNA extract was calculated for the infected bird groups from day 0 (pre-infection) until day 8 post-infection. [file Image3.jpeg]

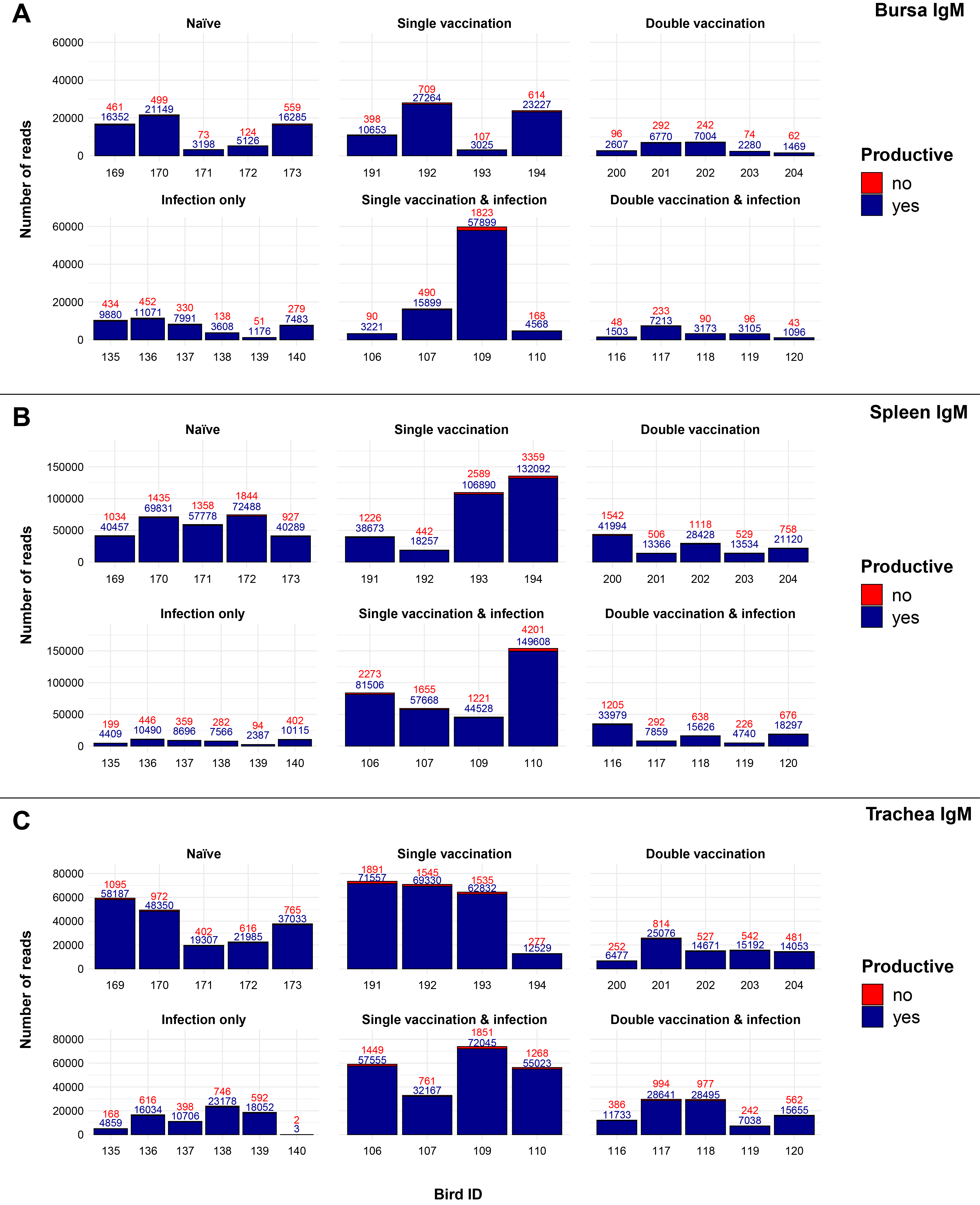

Supplement: Supplementary Figure 4 — Total number of IgM sequence reads identified in tissues of chickens that were subjected to different immunisation regimes. (A) Splenic samples, (B) bursal samples, (C) tracheal samples. Bird numbers displayed on the x axis and individuals are grouped based on the corresponding immunisation status which is illustrated above each panel. Productive and unproductive reads are shown in blue and red, respectively. [file Image4.jpeg]

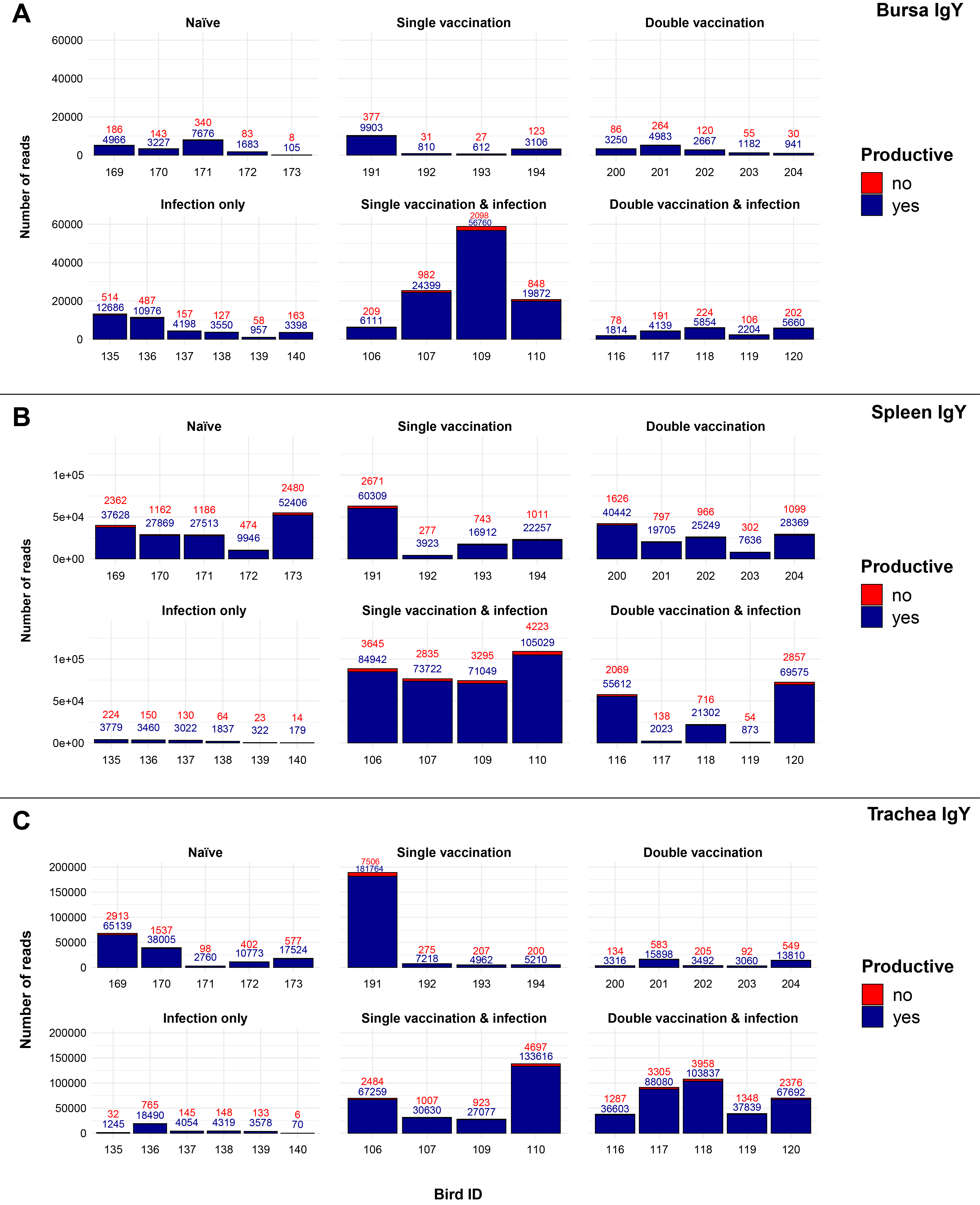

Supplement: Supplementary Figure 5 — Total number of IgY sequence reads identified in tissues of chickens that were subjected to different immunisation regimes. (A) Splenic samples, (B) bursal samples, (C) tracheal samples. Bird numbers displayed on the x axis and individuals are grouped based on the corresponding immunisation status which is illustrated above each panel. Productive and unproductive reads are shown in blue and red, respectively. [file Image5.jpeg]
