# Supplementary material for: Immunisation of chickens with inactivated and/or infectious H9N2 avian influenza virus leads to differential immune B-cell repertoire development
Source: Front Immunol. 2024 Oct 28;15:1461678. doi: 10.3389/fimmu.2024.1461678 (PMC11555566; doi:10.3389/fimmu.2024.1461678)
Supplement: Supplementary file 6 [file Table1.docx]

**Supplementary Table 1:** *Unique IgM and IgY sequences (clones) identified within the analysed samples*. Unique Clones were defined based on 100% CDR3 nucleotide identity.

| **Bird** | **Status** | **Tissue** | **Unique IgM Sequences** | **Unique IgY Sequences** |
| --- | --- | --- | --- | --- |
| 200 | Double vaccination | Bursa | 2377 | 2221 |
| 201 | Double vaccination | Bursa | 5395 | 2040 |
| 202 | Double vaccination | Bursa | 4574 | 1218 |
| 203 | Double vaccination | Bursa | 1904 | 615 |
| 204 | Double vaccination | Bursa | 1289 | 581 |
| 116 | Double vaccination & infection | Bursa | 1354 | 633 |
| 117 | Double vaccination & infection | Bursa | 6433 | 2175 |
| 118 | Double vaccination & infection | Bursa | 2094 | 1443 |
| 119 | Double vaccination & infection | Bursa | 2256 | 884 |
| 120 | Double vaccination & infection | Bursa | 786 | 1594 |
| 135 | Infection only | Bursa | 9074 | 6949 |
| 136 | Infection only | Bursa | 9944 | 6508 |
| 137 | Infection only | Bursa | 7435 | 3275 |
| 138 | Infection only | Bursa | 3103 | 2283 |
| 139 | Infection only | Bursa | 908 | 523 |
| 140 | Infection only | Bursa | 5806 | 2350 |
| 169 | Naïve | Bursa | 13783 | 1880 |
| 170 | Naïve | Bursa | 18520 | 2056 |
| 171 | Naïve | Bursa | 2481 | 1922 |
| 172 | Naïve | Bursa | 4058 | 869 |
| 173 | Naïve | Bursa | 6577 | N/A |
| 191 | Single vaccination | Bursa | 9028 | 4824 |
| 192 | Single vaccination | Bursa | 21107 | 689 |
| 193 | Single vaccination | Bursa | 2498 | 484 |
| 194 | Single vaccination | Bursa | 17799 | 1900 |
| 106 | Single vaccination & infection | Bursa | 2456 | 1945 |
| 107 | Single vaccination & infection | Bursa | 11879 | 8301 |
| 109 | Single vaccination & infection | Bursa | 36803 | 15606 |
| 110 | Single vaccination & infection | Bursa | 3039 | 5509 |
| 200 | Double vaccination | Spleen | 23110 | 11356 |
| 201 | Double vaccination | Spleen | 9066 | 6236 |
| 202 | Double vaccination | Spleen | 16812 | 8143 |
| 203 | Double vaccination | Spleen | 9568 | 3264 |
| 204 | Double vaccination | Spleen | 13516 | 7708 |
| 116 | Double vaccination & infection | Spleen | 19236 | 11899 |
| 117 | Double vaccination & infection | Spleen | 5515 | 653 |
| 118 | Double vaccination & infection | Spleen | 9762 | 4349 |
| 119 | Double vaccination & infection | Spleen | 3654 | 439 |
| 120 | Double vaccination & infection | Spleen | 10578 | 10876 |
| 135 | Infection only | Spleen | 3309 | 1327 |
| 136 | Infection only | Spleen | 6616 | 1880 |
| 137 | Infection only | Spleen | 6348 | 1660 |
| 138 | Infection only | Spleen | 5498 | 1136 |
| 139 | Infection only | Spleen | 1791 | N/A |
| 140 | Infection only | Spleen | 3849 | N/A |
| 169 | Naïve | Spleen | 20868 | 7218 |
| 170 | Naïve | Spleen | 32487 | 8075 |
| 171 | Naïve | Spleen | 25680 | 8548 |
| 172 | Naïve | Spleen | 29567 | 3288 |
| 173 | Naïve | Spleen | 21018 | 14324 |
| 191 | Single vaccination | Spleen | 24355 | 15560 |
| 192 | Single vaccination | Spleen | 8339 | 1753 |
| 193 | Single vaccination | Spleen | 49499 | 6424 |
| 194 | Single vaccination | Spleen | 63086 | 8463 |
| 106 | Single vaccination & infection | Spleen | 33609 | 18002 |
| 107 | Single vaccination & infection | Spleen | 26988 | 16030 |
| 109 | Single vaccination & infection | Spleen | 17090 | 14077 |
| 110 | Single vaccination & infection | Spleen | 50237 | 20084 |
| 200 | Double vaccination | Trachea | 3194 | 1256 |
| 201 | Double vaccination | Trachea | 10231 | 4361 |
| 202 | Double vaccination | Trachea | 6348 | 1393 |
| 203 | Double vaccination | Trachea | 6293 | 1092 |
| 204 | Double vaccination | Trachea | 6454 | 4235 |
| 116 | Double vaccination & infection | Trachea | 6136 | 6602 |
| 117 | Double vaccination & infection | Trachea | 12429 | 13400 |
| 118 | Double vaccination & infection | Trachea | 12759 | 16114 |
| 119 | Double vaccination & infection | Trachea | 2804 | 7039 |
| 120 | Double vaccination & infection | Trachea | 7704 | 12104 |
| 135 | Infection only | Trachea | 2240 | 565 |
| 136 | Infection only | Trachea | 6006 | 4323 |
| 137 | Infection only | Trachea | 4804 | 1582 |
| 138 | Infection only | Trachea | 8227 | 1617 |
| 139 | Infection only | Trachea | 7018 | 1539 |
| 169 | Naïve | Trachea | 13896 | 8822 |
| 170 | Naïve | Trachea | 10815 | 6517 |
| 171 | Naïve | Trachea | 4378 | 622 |
| 172 | Naïve | Trachea | 5644 | 1826 |
| 173 | Naïve | Trachea | 8841 | 3279 |
| 191 | Single vaccination | Trachea | 23110 | 23448 |
| 192 | Single vaccination | Trachea | 23760 | 2606 |
| 193 | Single vaccination | Trachea | 21677 | 1947 |
| 194 | Single vaccination | Trachea | 5390 | 1683 |
| 106 | Single vaccination & infection | Trachea | 20765 | 14237 |
| 107 | Single vaccination & infection | Trachea | 12865 | 7380 |
| 109 | Single vaccination & infection | Trachea | 19071 | 6069 |
| 110 | Single vaccination & infection | Trachea | 15541 | 21074 |
